# Research on Establishment of Abnormal Phlegmatic Syndrome with Premature Ovarian Failure Rat Model and Effects of Balgham Munziq Treatment

**DOI:** 10.1155/2018/3858209

**Published:** 2018-03-26

**Authors:** Muladili Yuemaier, Mayire Tuerhong, Abulizi Keremu, Nafeisha Kadeer, Ayiguzhali Aimaiti, Xilinguli Wushouer, Adilijiang Yiming, Xiamixinuer Yilike

**Affiliations:** ^1^Department of Biology, School of Medicine, Xinjiang Medical University, Ürümqi, Xinjiang 830011, China; ^2^Morphology Center, School of Medicine, Xinjiang Medical University, Ürümqi, Xinjiang 830000, China; ^3^Department of Human Anatomy, School of Medicine, Xinjiang Medical University, Ürümqi, Xinjiang 830011, China

## Abstract

This study aimed to establish and explore the biological basis of abnormal phlegmatic syndrome with premature ovarian failure (POF) model in rats based on the Uighur medicine (UM) in the first place and investigate the effects of unique herbal medicine, Balgham Munziq (BMq). Mature female Wistar rats were fed with spinach and coriander in cold and humid condition for approximately 20 weeks until abnormal phlegmatic syndrome (APS) model was established. When APS model was confirmed by Uighur medical experts, APS with POF disease rats were subdivided into APS with POF disease model group and APS with POF disease treated with BMq group; the rest of them were subdivided into APS model group and APS treated with BMq group. The results show that biological characteristics of animals in the course of modeling period were in accordance with clinical features of abnormal phlegmatic syndrome (APS) in Uighur medicine. Herbal medicine BMq not only reverted reproductive hormone levels disorders but also improved the function of hypothalamic-pituitary-ovarian axis and regulated secretion of monoamine neurotransmitters. APS is most likely to cause pathological changes of hypothalamic-pituitary-ovarian axis and lead to the occurrence of POF and BMq is effective in the treatment of APS with POF disease.

## 1. Introduction

Premature ovarian failure (POF) is becoming more and more prevalent among the ladies who are below the age of 40 [1], which is also called premature menopause or primary ovarian insufficiency [2]. The previous studies indicate that approximately 2-3% of young women suffer from POF caused by unknown cases [3]. POF shows clinical features such as premature menopause, infertility, and ovarian insufficiency which are accompanied by particular symptoms like vaginal dryness, hot flashes, hyposexuality, and so on [4]. There are many factors to cause POF; the previous researches indicate that chromosomal or genetic abnormalities, autoimmunity, infections, enzymatic or metabolic factors, environmental toxins, and iatrogenic influences are involved [5, 6]. The current consensus of opinion is that psychological stress, such as chronic anxiety, sadness, depression, or other negative emotions, can also cause POF by changing the physiology function of the hypothalamic-pituitary-target gland axis, which results in the occurrence of the hypothalamic-pituitary-ovarian axis disorder [7, 8].

POF can be diagnosed by the determination of an elevated serum level of FSH (FSH > 40 IU/L) in clinical detection. Besides, echography such as ovarian ultrasonic testing is also used as assistant diagnosis in POF [9, 10]. The most recent studies show that the new indicators were found to detect the occurrence of POF such as Anti-Müllerian hormone (AMH) which could be used as new biomarkers in this process. AMH has a paracrine role in the ovary; the advantage of AMH as an ideal biomarker is dependent on the negative feedback of the gonadotropins [11, 12].

Speaking of which, management of POF in western medicine nowadays involves immunosuppressive treatment, hormone therapy such as hormone replacement therapy (HRT), and ovarian transplantation [13, 14]. Immunosuppressive treatment presents possibility of resumption of ovarian function; nevertheless, treatment with immunosuppressive agents failed to reverse the course of the ovarian autoimmunity or enhance the ovarian response to gonadotropins [15]. A large number of studies show that hormone therapy improves the quality of distressful life and reduces the severe consequences in women who suffered from POF [16]. The ovarian transplantation is doable, but due to the destruction of gonads in the course of treatment preparing for transplantation or the emergence of rejection, it is not widely chosen in the clinical treatments. Even if it is, POF patients who suffered from ovarian transplantation treatment would enter the premature menopause earlier [17].

Uighur medicine (UM) is the most important part of the traditional Chinese medicine which has already existed for thousands of years [18]. Hilit theory is the core of the UM; it claims that human body is composed of four kinds of Hilits: Sapra, Kan, Sawda, and Balgham (phlegm). They keep homeostasis in our body in quantitative and qualitative way. Meanwhile, those four Hilits also maintain the normal physiological status of the body. Nonetheless, once the balance between Hilits is broken, this would cause many different diseases [19, 20]. BMq, a traditional Uighur herbal medicine recipe, has been widely used in UM for a long time [21]. It could eliminate the Aabnormal Balgham Hilit from the body and restore the balance between all Hilits.

Despite the fact that BMq has been playing an effective role in the management of the diseases caused by APS, the molecular mechanism of BMq is not fully understood. To study APS with POF disease, we have established APS with POF rat model based on the UM in the first place to find out the biological basis in animal models.

## 2. Materials and Methods

### 2.1. Animals

90 mature female Wistar rats that were verified by vaginal smear test were provided from the Laboratory Animal Centre of Xinjiang Medical University (certificate number: SCXK (Xin) 2011–0004) and weighed 180 ± 20 g. These rats were kept in the clean standardized laboratory condition, with illumination from 8:00 AM to 8:00 PM, at room temperature of 25 ± 2°C and humidity of 55 ± 5% for three to five days so that they could fit in the experimental condition. During the course of study, the rats were allowed food and water ad libitum. All experiments were approved by the Ethics Committee of the Xinjiang Medical University, which were in accordance with the relevant national guidelines, including any relevant details.

### 2.2. Chemicals and Reagents

BMq (mixture, 500 ml) was purchased from Xinjiang Uighur Medical Hospital (Ürümqi, China). Spinach and coriander seeds were purchased from Xinjiang Western Seed Industry Co., Ltd. (Ürümqi, China). Cisplatin was from Sigma Chemical Co. (St. Louis, MO, USA). Radioimmunoassay kits for rat estradiol 2 (E2), follicle stimulating hormone (FSH), and luteinizing hormone (LH) were provided by Beijing Northern Biotechnology Institute (Beijing, China). ELISA kits for E2, FSH, LH, prolactin (PRL), progesterone (P), and inhibin B (INHB) were obtained from Elabscience Biotechnology Co., Ltd. (Wuhan, China).

### 2.3. Treatments

All of 90 rats were divided randomly into APS model group (*n* = 70), control group (*n* = 10), and cisplatin-induced POF group (*n* = 10). Control group rats were raised at normal condition with temperature of 25 ± 2°C and humidity of 55 ± 5% and fed with normal fodder throughout the study. The APS group rats were raised in animal facility at temperature of 6 ± 1°C and humidity of 80~90% from 8 AM to 8 PM and fed with cold natural fodder (the ratio of normal fodder, spinach, and coriander seeds was 7 : 1.5 : 1.5) which was especially made. Both groups were modeled for approximately 20 weeks until the APS was established. Cisplatin-induced POF group rats were injected with cisplatin for 10 days at the dose of 1.5 ml/kg [4]. Then blood was plucked from all rats by orbital vein plexus to measure the serum level of E2, FSH, and LH by radioimmunoassay. According to the above parameters, estrous cycle, and quantitative indicators of biological characterization of each rat, APS rats with POF disease were subdivided into APS with POF disease group (B1), BMq treatment of APS with POF disease group (B2), and natural treatment of APS with POF disease group (B3). The rest that were without POF disease were subdivided into APS group (A1), BMq treatment of APS group (A2), and natural treatment of APS group (A3). During the treatment, groups B1 and A1 were kept in a cold and humid condition as usual. Groups B2 and A2 were treated by intragastric administration of BMq 27 ml/kg a day according to human equivalent dose calculated by the method of Reagan-Shaw et al. [22] and fed with normal fodder in normal condition. Groups B3 and A3 were transferred to normal condition the same as the control group, which were set to prove that the stability of the model had been established.

### 2.4. Estrous Cycle and Biological Characterization

For the subsequent experiment, the estrous cycle of all rats was examined by vaginal smear test at 10 AM continuously. Vaginal smears were collected and smeared on glass slides by cotton swab which was dipped in 0.9% NaCl. After air-drying, the samples were stained with methylene blue in 9.5% ethanol, washed, covered with cover glass, and observed under microscope. Observation of estrous cycle was followed by proestrus, with 100% of intact live epithelial cells; estrus, with 100% of cornified epithelial cells; metestrus, with approximately 50% of cornified epithelial cells and 50% of leukocytes; and diestrus, with 80~100% of leukocytes [4]. The qualitative and quantitative indicators of biological characterization were determined per week during the study; for example, activities, stimulus response, and quantitative indicators were measured such as animal body weight, water intake, food intake, urine volume, and stool quantity; the most important dialectic parameters in the process of confirming the APS model were established based on Uighur medicine.

### 2.5. Determination of Serum Levels of Reproductive Hormones

For each animal, a part of blood was plucked from abdominal aorta at the end of experiment; the serum was isolated for determination of concentration levels of E2, FSH, LH, PRL, P, and INHB by enzyme-linked immunosorbent assay (ELISA) using ELISA kits followed by ELISA kit's specification.

### 2.6. Histological Examination

After all animals were sacrificed, the ovaries, hypothalami, and pituitaries of all rats were dissected and immersed in 4% neutral buffered paraformaldehyde for at least 48 h and then embedded in paraffin. The samples were sliced at 4 *μ*m thick sections. All of sections were stained with hematoxylin and eosin (H&E) and, subsequently, observed under microscope and photographed.

### 2.7. Measurement of Plasma Levels of Monoamine Neurotransmitters

Plasma was isolated immediately; the 400 *μ*L samples were pretreated with 250 *μ*L ammonium acetate (50 mM). 96-well extraction panel was pretreated with 200 *μ*L methanol and 200 *μ*L distilled water. Then plasma samples were added to extraction panels and washed successively with 200 *μ*L ammonium acetate (20 mM) and 200 *μ*L acetonitrile : isopropanol (50 : 50) solution. 96-well extraction panels were dried by nitrogen, and the solvent in the column bed was removed as much as possible. In the end, 50 *μ*L acetonitrile with 2% formic acid : distilled water (85 : 15) solution was used to elute the target compound from 96-well extraction panels to sample collection board. The plasma samples and standard substance concentration levels of monoamine neurotransmitters such as norepinephrine (NE), epinephrine (EP), dopamine (DA), and 5-hydroxytryptamine (5-HT) were measured by ultra-performance liquid chromatography-mass spectrometry (UPLC-MS/MS); the value of certain concentration was calculated according to the standard curve for each animal.

### 2.8. Statistical Analysis

All of the results were analyzed by SPSS 22.0 (SPSS Inc., Chicago, IL, USA) via one-way analysis of variance (ANOVA) and presented as mean ± SD ($\overset{-}{x}\pm s$); Student-Newman-Keuls test was performed for comparisons between groups. *P* < 0.05 was considered statistically significant.

## 3. Results

### 3.1. Changes in General States

**Figure 1:**
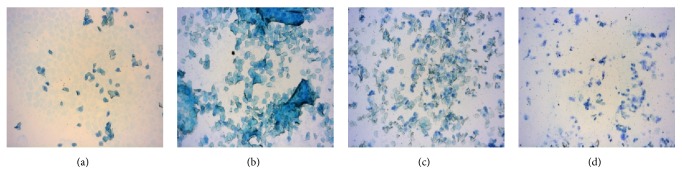
Changes of estrous cycle. (a) Proestrus; (b) estrus; (c) metestrus; (d) diestrus. Magnification: ×100.

**Figure 2:**
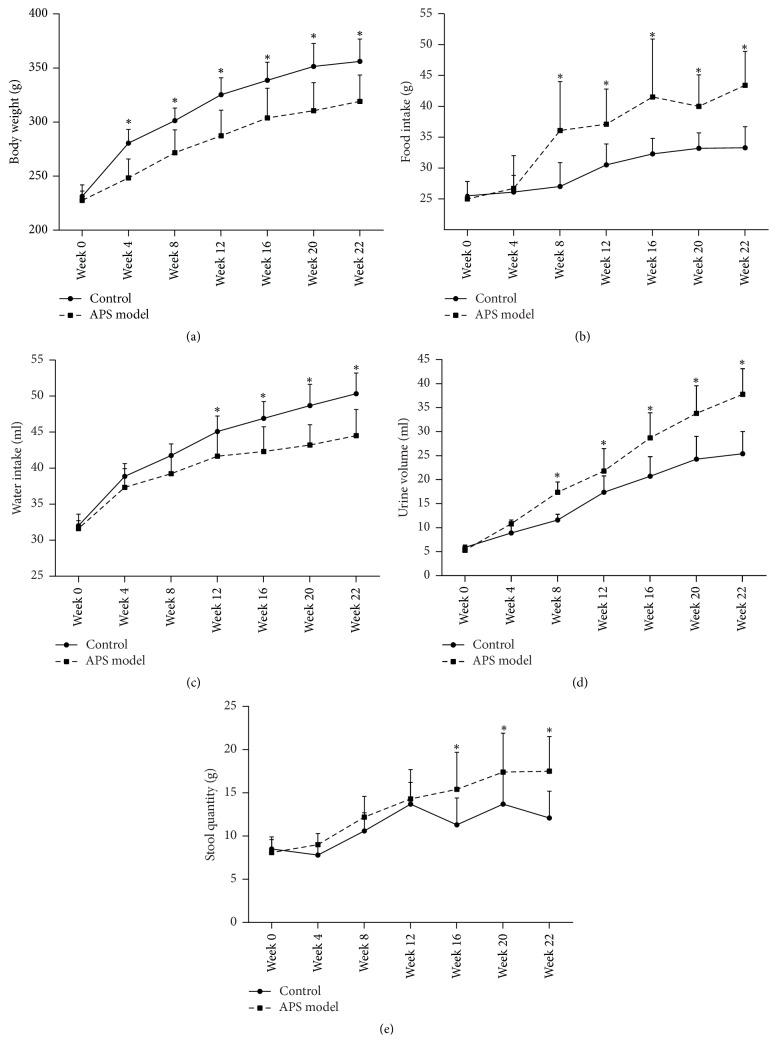
Changes of biological characterizations: body weight (a), food intake (b), water intake (c), urine volume (d), and stool quantity (e) during modeling period. Asterisks show significant differences when APS model group was compared with control group in different time period (^*∗*^*P* < 0.05; mean ± SD).

Control group rats showed four states of estrous cycle as shown in Figure 1, whereas APS model group displayed longer estrous cycle, approximately 18.6% (13 of 70) of which exhibited estrous cycle disorders without proestrus and estrus until APS model was established. Results of biological characterizations showed that APS model group rats not only had less activity and hyposensitivity to external stimulus but also showed retarded growth and decrease in water intake. Meanwhile, APS model group showed an increase in food intake, urine volume, and stool quantity compared with the control group (Figure 2). All results of characterizations shown above were in accordance with clinical features of APS in Uighur medicine.

### 3.2. Changes of Serum Levels of Relative Hormones in Each Group after BMq Treatment

All of the BMq treatment periods were done; the serum levels of E2, FSH, LH, PRL, P, and INHB were measured and listed in Table 1. Two animals in group B2 and one animal in POF model group died from unknown reasons. As is shown, control group rats had low FSH and LH concentration level but a high concentration level of E2. On the other side, A1, B1, and POF model groups were manifested with the significantly higher levels of FSH and LH; meanwhile, serum level of E2 was decreased. PRL level showed no significant differences between groups except POF group which was compared with the control, although there were no big changes. Serum levels of P and INHB were significantly decreased in groups A1, B1, and POF which were compared with control group.

Groups A2 and B2 were treated with BMq, and it was shown that levels of relative hormones were retrieved but still had difference compared to control group. Groups A3 and B3 showed no significant differences compared with A2 and A3 which manifested that APS model was relatively stable in the normal condition when the model was established.

### 3.3. Morphological Changes of Hypothalamic-Pituitary-Ovarian Axis

**Figure 3:**
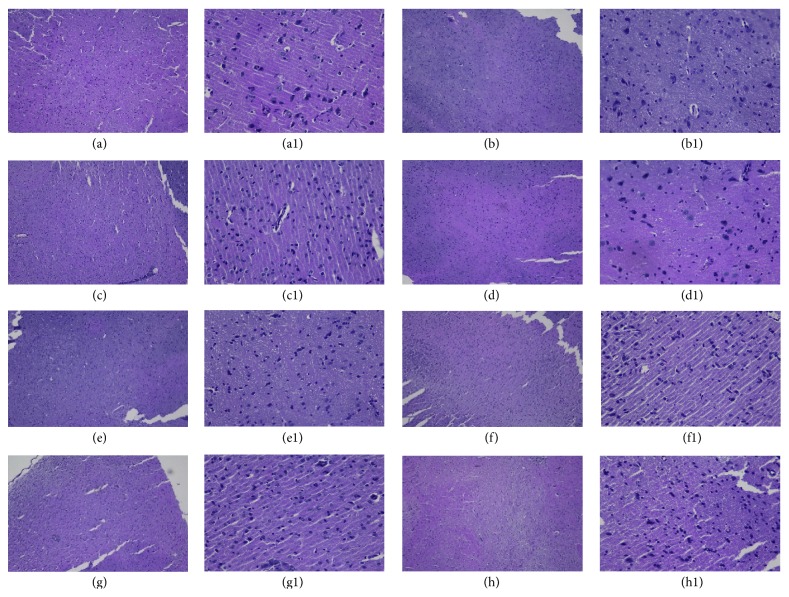
Histology of hypothalami from each group: all sections were stained with H&E. (a), (a1): control; (b), (b1): POF model group; (c), (c1): group A1; (d), (d1): group A2; (e), (e1): group A3; (f), (f1): group B1; (g), (g1): group B2; (h), (h1): group B3. ((a)–(h)) Magnification: ×100. ((a1)–(h1)) Magnification: ×400.

Hypothalamic-pituitary-ovarian axis is involved in adjustment of the ovarian function by regulating reproductive hormones. In this study, hematoxylin-eosin staining (H&E staining) was performed to examine morphological changes of hypothalamus, pituitary, and ovary sections. In hypothalamic sections (Figure 3), it can be observed that large amounts of glial cells and neuron were well distributed with their complete structure, without edema as well in the control group (Figure 3(a)). Pathological changes such as varying degrees of loosing of hypothalamic matrix, decreasing number of neuronal cells without complete structure, and intercellular substance edema were observed in the POF model, A1, A3, B1, and B3 groups (Figures 5(b), 5(c), 5(e), 5(f), and 5(h)).

**Figure 4:**
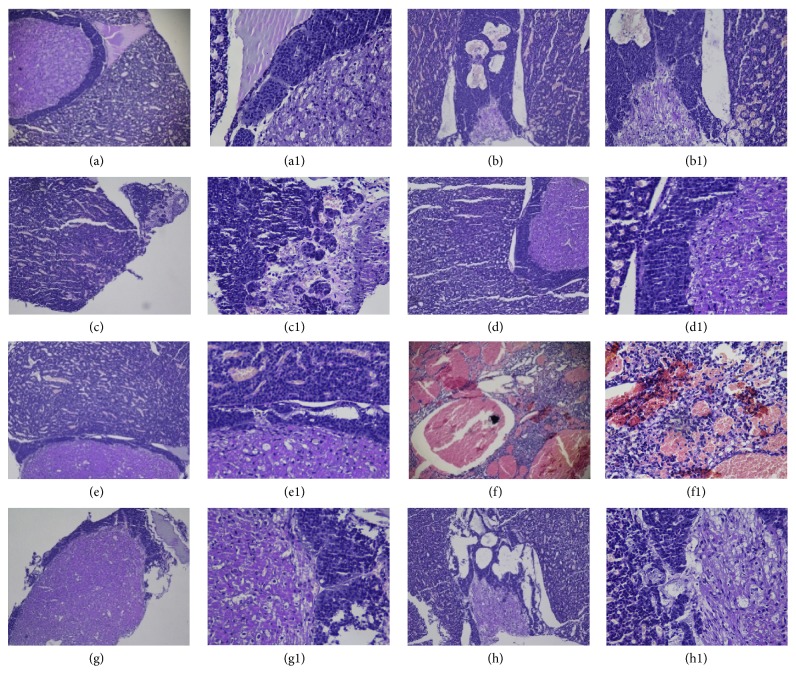
Histology of pituitaries from each group: all sections were stained with H&E. (a), (a1): control; (b), (b1): POF model group; (c), (c1): group A1; (d), (d1): group A2; (e), (e1): group A3; (f), (f1): group B1; (g), (g1): group B2; (h), (h1): group B3. ((a)–(h)) Magnification: ×100. ((a1)–(h1)) Magnification: ×400.

In pituitary sections (Figure 4), tight pituitary substrate, normal structure of eosinophil and chromophobe cells with round shape or polygon shape, abundant sinusoids, and deeper stained nucleus were observed in control group (Figure 4(a)). Pathologic changes such as loose structure of neurohypophysis, unclear circumscription between nucleus and cytoplasm, and cellular edema occurred in APS model group rats instead.

**Figure 5:**
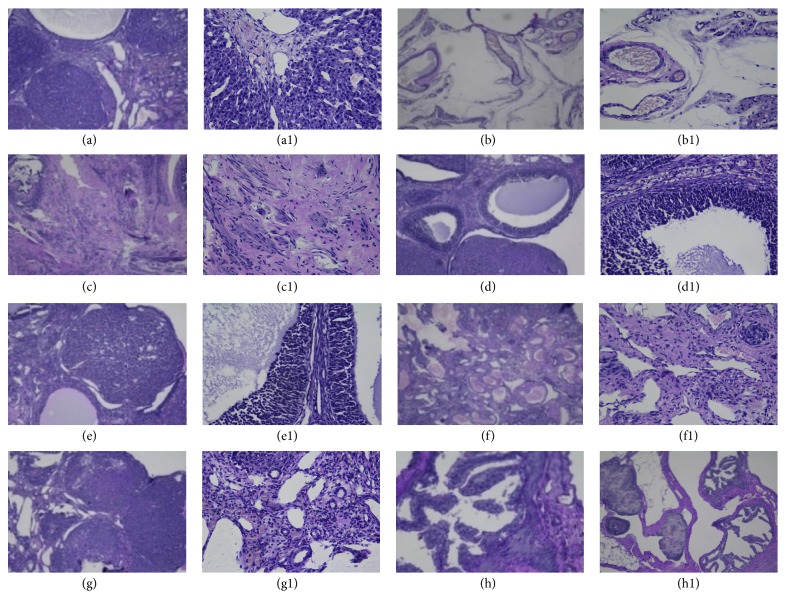
Histology of ovaries from each group: all sections were stained with H&E. (a) control; (b) POF model group; (c) group A1; (d) group A2; (e) group A3; (f) group B1; (g) group B2; (h) group B3. Magnification: ×400.

Ovarian sections (Figure 5) from control group (Figure 5(a)) showed that each stage of follicles such as primary follicles, secondary follicles, growing follicles, and a large amount of mature follicles could be seen. Meanwhile, pathological changes such as lack of complete corpus luteum, increasing amount of atretic follicle, various degrees of cellular edema, and occurrence of fibrosis were observed in POF model, A1, A3, B1, and B3 groups (Figures 5(b), 5(c), 5(e), 5(f), and 5(h)). After BMq treatment, increased number of mature follicles and amount of corpus luteum and decreased follicular atresia were observed in groups A2 and B2 (Figures 5(d) and 5(g)).

### 3.4. Changes of Plasma Levels of Monoamine Neurotransmitters

**Figure 6:**
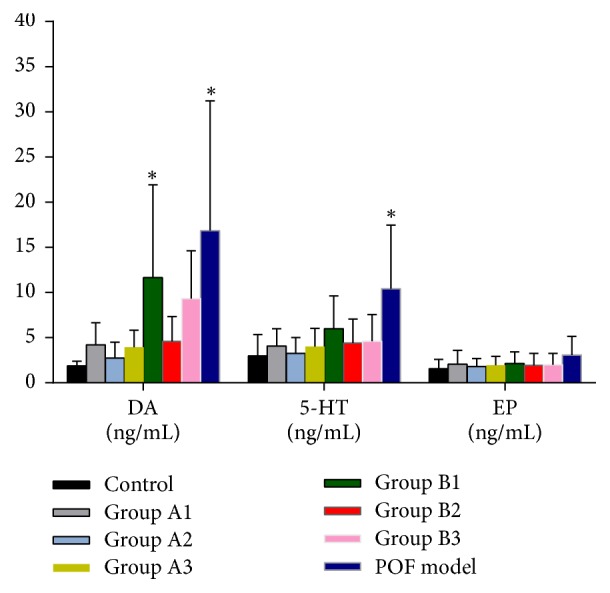
Plasma levels of DA, 5-HT, and EP in each group after BMq treatment. Asterisks show significant differences in all model groups versus the control group (^*∗*^*P* < 0.05; mean ± SD).

Ultra-performance liquid chromatography-mass spectrometry (UPLC-MS/MS) was used to determine the plasma levels of norepinephrine (NE), epinephrine (EP), dopamine (DA), and 5-hydroxytryptamine (5-HT). The results indicate that DA levels in plasma were gradually increased in groups A1 and B1 and POF model group compared with the control; however, there was no significant difference between group A1 and control group. Plasma levels of 5-HT were upregulated in the modeling groups; nonetheless, there were no significant differences compared with the control group except in POF model group. The EP levels in plasma showed an increasing trend but there were no significant differences between groups (all *P* > 0.05). Unfortunately, plasma levels of NE in most of animals could not be determined so that subsequent data analyses were not able to be performed. In addition, groups A2 and B2 which were treated with BMq could decrease the plasma levels of DA, 5-HT, and EP in different degrees (Figure 6).

## 4. Discussion

Nowadays, POF shows a prevalent trend by the developing of our modern society, which is also a common condition that can bring negative effects to women's physiological or mental health [16]. Studies indicate that there are several possible factors which may be associated with the occurrence of POF. Genetic basis may be involved; however, only a small proportion of genes which are influencing idiopathic POF have been confirmed so far; it would take more novel molecular pathways to reveal the molecular mechanisms and heritability of POF. Additionally, autoimmunity, iatrogenic causes, and metabolic disorders are also considered as the mainly causes of POF disease [23]. Some suggest that ovarian function is influenced by living habits, psychological changes, or environmental factors; there is no effective therapy for POF because of miscellaneous causes [24].

APS with POF is a systemic disease according to the traditional Uighur medicine, which mostly occurs due to living habits such as eating cold and humid natural food and exposure to the cold and humid natural environment for a long time. Abnormal Balgham Hilit is abnormally exuberated in this chronic process so that body heat is decreased, associated with low metabolic activity and metabolites stack within tissues. Subsequently, early pathological changes are caused and imbalanced Hilits in the body would lead to multiple diseases that include POF. The APS rat model that had been established is in accordance with clinical features of APS patients in traditional Uighur medicine and shows that modeling concept is feasible. A3 and B3 model groups that were kept in natural condition exhibited mostly the same characteristics as A2 and B2 in all of parameters that have been measured, which demonstrates that APS rat model and APS with POF disease rat model are stable. BMq, a Uighur herbal medicine formula, could restore the balance between all four Hilits by eliminating the extra abnormal phlegm. The underlying mechanisms were trying to be investigated by performing BMq treatment of APS with POF model rats in this study. The study indicates that BMq not only reverts estrous cycle but also adjusts the biological features such as decreasing food intake, increasing water intake, and promoting normal growth of body.

As is all known, FSH plays significant role in the follicular development, which participates in recruitment and selection of follicles and development of dominant follicles and all stages of maturity. As a biomarker, INHB is thought to directly reflect the function of granule cells; besides, INHB is associated with secretion of FSH of negative feedback from ovary to pituitary [25]. Our study showed that serum levels of E2 and INHB were decreased in groups A1 and B1 and POF model group; long stay in cold and humid condition is most likely to damage the ovarian and pituitary morphological forms, therefore decreasing the quantity of granulosa cells; synthesis of INHB is retarded, which subsequently reduces the negative feedback regulation; thus, levels of gonadotropin are upregulated. Study showed that there was no significant difference between A1 and control in serum level of E2, while serum level of INHB showed significant difference instead, which indicates that APS has potentiality to develop into POF.

Hypothalamic-pituitary-ovarian axis is the most important part of integral reproductive endocrine system in women [26]. Gonadotropin-releasing hormone (GnRH) secreted by hypothalamus regulates the pituitary to release gonadotropin and secretion of sex hormones in ovary. Emergence of pathological changes of gonadal axis was most likely to cause reproductive hormones disorder in APS with POF disease. After BMq treatment, not only the clinical indicators such as E2, FSH, and INHB in peripheral blood but also the histomorphology changes of A1 and B1 were improved. Research indicates that BMq can regulate the reproductive hormones level; additionally, it also can improve ovarian function and increase the mature follicles quantity and number of granule cells, suggesting that BMq might remit the POF-caused pathogenic condition by improving the physiological function of hypothalamic-pituitary-ovarian axis.

Monoamine neurotransmitters contain DA, 5-HT, NE, and EP which are extremely unstable in the air and could easily react with air and cause oxidative deterioration to lose efficacy. In consideration of multiple interfering substances and their low content in blood, UPLC-MS/MS was used to determine the concentration levels of monoamine neurotransmitters in plasma. Results showed that plasma levels of DA, 5-HT, and EP were increased; only the DA levels in APS with POF disease model (B1) and POF model had significant differences with control; increasing trend could be seen in 5-HT and EP, but there was no significant difference between B1 and control. Unfortunately, plasma level of NE could not be measured in most of samples; the following possible reasons were considered: firstly, blood samples might have been degraded; secondly, concentration level of NE was lower than detection limit. The second explanation was preferred. DA is an important part in central nervous system; previous studies have demonstrated that DA's function disorder is associated with schizophrenia and Parkinson disease or some other mental abnormality [27]. In the chronic cold and humid stress condition, not only the pituitary-adrenal cortex system but also the sympathetic adrenomedullary system is involved; thus, contents of catecholamine are elevated in blood; besides, DA is precursor of NE [28]. In conclusion, long-term exposure to cold and humid stress is most likely to activate hypothalamic-pituitary-ovarian axis and sympathetic adrenomedullary system at a chronic excitatory state and increase the levels of monoamine neurotransmitters such as DA, NE, and EP which inhibit hypothalamus-pituitary-genital axis, while generating endocrine and immune function disorder [29, 30], eventually exceeding body's autoregulation range, and it leads to ovarian failure. BMq may revert this process to regulate the monoamine neurotransmitters levels and improve the ovarian function.

Several limitations should be declared regarding the current study. First of all, establishment of APS rat model was based on the clinical Uighur medicine; in addition, it was the first time to put forward the opinion of APS with POF disease; thus, it should be verified with repeated trials in a long term. Secondly, BMq is the unique Uighur herbal recipe for diseases caused by APS; after BMq treatment, pathologic changes that occurred in APS with POF disease model group rats were improved in some degree. However, because of the complex molecular mechanisms of POF, specific pharmacological mechanism of BMq needs to be cleared in a further study.

## 5. Conclusions

In conclusion, the current study shows that occurrence of APS with POF disease not only may be caused by recession of ovarian function but also may be associated with pathological changes of gonadal axis. Uighur herbal medicine BMq is effective for the treatment of APS with POF; its mechanism of action might be associated with the improvement of pathological changes in gonadal axis.

## Figures and Tables

**Table 1:** Serum levels of relative hormones in each group after treatment with BMq.

Group (*N*)	E_2_/(pg/mL)	FSH/(mlU/mL)	LH/(mlU/mL)	PRL/(pg/mL)	P/(mlU/mL)	INHB/(pg/mL)
Control (10)	49.17 ± 5.87	2.34 ± 0.67	3.72 ± 1.00	27.49 ± 9.36	6.64 ± 2.16	191.67 ± 36.11
A1 (10)	46.53 ± 4.63	3.78 ± 0.59^*∗*^	4.58 ± 1.16	27.48 ± 10.86	4.90 ± 1.82^*∗*^	150.43 ± 19.80^*∗*^
A2 (15)	44.69 ± 8.22	2.77 ± 1.05^#^	4.03 ± 0.59	28.57 ± 8.00	5.43 ± 1.96	183.19 ± 29.22^#^
A3 (10)	44.89 ± 4.65	3.54 ± 0.87	4.23 ± 0.91	27.03 ± 7.70	4.71 ± 1.13	157.82 ± 32.39
B1 (10)	21.09 ± 7.80^*∗*#^	4.57 ± 0.89^*∗*#^	5.84 ± 1.51^*∗*#^	27.83 ± 6.35	3.84 ± 1.45^*∗*#^	105.66 ± 32.51^*∗*#^
B2 (13)	38.81 ± 6.11^▽^	2.14 ± 0.35^▽^	4.76 ± 0.45	32.43 ± 3.99	5.46 ± 1.04^▽^	189.61 ± 34.91^▽^
B3 (10)	25.97 ± 5.56	4.08 ± 1.20	5.02 ± 1.17	28.00 ± 6.64	4.13 ± 1.99	128.45 ± 53.08
POF model (9)	18.85 ± 9.97^*∗*^	4.46 ± 0.92^*∗*^	6.16 ± 1.64^*∗*^	25.69 ± 11.02^*∗*^	4.36 ± 1.90^*∗*^	89.14 ± 21.16^*∗*^

(a) All data are presented as mean ± SD; *N* means number of animals; (b) ^*∗*^*P* < 0.05 versus the control group; ^#^*P* < 0.05 versus group A1; ^▽^*P* < 0.05 versus group B1.
